# Supplementary material for: Criteria to define rare diseases and orphan drugs: a systematic review protocol
Source: BMJ Open. 2022 Jul 29;12(7):e062126. doi: 10.1136/bmjopen-2022-062126 (PMC9345065; doi:10.1136/bmjopen-2022-062126)
Supplement: Supplementary data [file bmjopen-2022-062126supp001.pdf]

Appendix 1: Search strategy

Research question: What are the criteria to define Rare Diseases and Orphan Drugs globally?

	PubMed	Medline	Embase	Scopus	WOS
Concept 1 (Criteria)	Criteria [All Fields] OR Standard*[All Fields] OR classification [All Fields] OR Measure*[All Fields] OR Condition*[All Fields] OR Principle*[All Fields] OR Requirement*[All Fields] OR Scale*[All Fields] OR Parameter*[All Fields] OR Indicator*[All Fields] OR Norm*[All Fields]	(Criteria or Standard* or classification or Measure* or Condition* or Principle* or Requirement* or Scale* or Parameter* or Indicator* or Norm*).mp. [mp=title, abstract, original title, name of substance word, subject heading word, floating sub-heading word, keyword heading word, organism supplementary concept word, protocol supplementary concept word, rare disease supplementary concept word, unique identifier, synonyms]	(Criteria or Standard* or classification or Measure* or Condition* or Principle* or Requirement* or Scale* or Parameter* or Indicator* or Norm*).mp. [mp=title, abstract, heading word, drug trade name, original title, device manufacturer, drug manufacturer, device trade name, keyword, floating subheading word, candidate term word]	TITLE-ABS-KEY (criteria OR standard* OR classification OR measure* OR condition* OR principle* OR requirement* OR scale* OR parameter* OR indicator* OR norm*)	ALL FIELDS: (criteria OR standard* OR classification OR measure* OR condition* OR principle* OR requirement* OR scale* OR parameter* OR indicator* OR norm*) Timespan: All years. Indexes: SCI-EXPANDED, SSCI, A&HCI, CPCI-S, CPCI-SSH, ESCI.
Concept 2 (Define)	Defin*[All Fields] OR Mean*[All Fields] OR Description [All Fields] OR Character*[All Fields] OR Explan*[All Fields] OR delineate [All Fields] OR detail [All Fields] OR interpret[All Fields] OR determine[All Fields] OR elucidate[All Fields] OR illustrate[All Fields] OR exemplify[All Fields]	(Defin* or Mean* or Description or Character* or Explan* or delineate or detail or interpret or determine or elucidate or illustrate or exemplify).mp. [mp=title, abstract, original title, name of substance word, subject heading word, floating sub-heading word, keyword heading word, organism supplementary concept word, protocol supplementary concept word, rare disease supplementary concept word, unique identifier, synonyms]	(Defin* or Mean* or Description or Character* or Explan* or delineate or detail or interpret or determine or elucidate or illustrate or exemplify).mp. [mp=title, abstract, heading word, drug trade name, original title, device manufacturer, drug manufacturer, device trade name, keyword, floating subheading word, candidate term word]	TITLE-ABS-KEY (defin* OR mean* OR description OR character* OR explan* OR delineate OR detail OR interpret OR determine OR elucidate OR illustrate OR exemplify)	ALL FIELDS: (defin* OR mean* OR description OR character* OR explan* OR delineate OR detail OR interpret OR determine OR elucidate OR illustrate OR exemplify) Timespan: All years. Indexes: SCI-EXPANDED, SSCI, A&HCI, CPCI-S, CPCI-SSH, ESCI.
Concept 3 (Rare Disease(s)	"Rare Diseases"[Mesh] OR “Orphan disease*”[All Fields] OR “Rare condition*”[All Fields] OR “Rare disorder*”[All Fields] OR “Rare disability*”[All Fields] OR “Neglected disease*”[All Fields] OR “Undiagnosed disease*”[All Fields] OR “Low-frequency disease*”[All Fields] OR “life-threatening disease*”[All Fields] OR “debilitating disease*”[All Fields] OR “severe disease*”[All Fields] OR “intractable disease*”[All Fields]	(Orphan disease* or Rare condition* or Rare disorder* or Rare disability* or Neglected disease* or Undiagnosed disease* or Low-frequency disease* or life-threatening disease* or debilitating disease* or severe disease* or intractable disease* or Rare Disease*).mp. [mp=title, abstract, original title, name of substance word, subject heading word, floating sub-heading word, keyword heading word, organism supplementary concept word, protocol supplementary concept word, rare disease supplementary concept word, unique identifier, synonyms]	(Orphan disease* or Rare condition* or Rare disorder* or Rare disability* or Neglected disease* or Undiagnosed disease* or Low-frequency disease* or life-threatening disease* or debilitating disease* or severe disease* or intractable disease* or Rare Disease*).mp. [mp=title, abstract, heading word, drug trade name, original title, device manufacturer, drug manufacturer, device trade name, keyword, floating subheading word, candidate term word]	TITLE-ABS-KEY ("Orphan disease*" OR "Rare condition*" OR "Rare disorder*" OR "Rare disability*" OR "Neglected disease*" OR "Undiagnosed disease*" OR "Low-frequency disease*" OR "life-threatening disease*" OR "debilitating disease*" OR "severe disease*" OR "intractable disease*" OR "Rare Disease*")	ALL FIELDS: ("Orphan disease*” OR “Rare condition*” OR “Rare disorder*” OR “Rare disability*” OR “Neglected disease*” OR “Undiagnosed disease*” OR “Low-frequency disease*” OR “life-threatening disease*” OR “debilitating disease*” OR “severe disease*” OR “intractable disease*” OR “Rare Disease*") Timespan: All years. Indexes: SCI-EXPANDED, SSCI, A&HCI, CPCI-S, CPCI-SSH, ESCI.

Abozaid GM, *et al. BMJ Open* 2022; 12:e062126. doi: 10.1136/bmjopen-2022-062126

Concept 4 (Orphan Drug(s))	"Orphan Drug Production"[Mesh] OR "Orphan medicinal product*" [All Fields] OR "Orphan product*"[All Fields] OR "Orphan subset*"[All Fields] OR "Orphan indication*"[All Fields] OR "Highly specialized technolog*"[All Fields] OR "Priority review drug*"[All Fields] OR "Orphan Drug*"[All Fields]	(Orphan medicinal product* or Orphan product* or Orphan subset* or Orphan indication* or Highly specialized technolog* or Priority review drug* or Orphan Drug* or Orphan Drug Production*).mp. [mp=title, abstract, original title, name of substance word, subject heading word, floating sub-heading word, keyword heading word, organism supplementary concept word, protocol supplementary concept word, rare disease supplementary concept word, unique identifier, synonyms]	(Orphan medicinal product* or Orphan product* or Orphan subset* or Orphan indication* or Highly specialized technolog* or Priority review drug* or Orphan Drug* or Orphan Drug Production*).mp. [mp=title, abstract, heading word, drug trade name, original title, device manufacturer, drug manufacturer, device trade name, keyword, floating subheading word, candidate term word]	TITLE-ABS-KEY ("Orphan medicinal product*" OR "Orphan product*" OR "Orphan subset*" OR "Orphan indication*" OR "Highly specialized technolog*" OR "Priority review drug*" OR "Orphan Drug Production*" OR "Orphan Drug*")	ALL FIELDS: ("Orphan medicinal product*" OR "Orphan product*" OR "Orphan subset*" OR "Orphan indication*" OR "Highly specialized technolog*" OR "Priority review drug*" OR "Orphan Drug Production*" OR "Orphan Drug*") Timespan: All years. Indexes: SCI-EXPANDED, SSCI, A&HCI, CPCI-S, CPCI-SSH, ESCI.
Total	(((Criteria [All Fields] OR Standard*[All Fields] OR classification [All Fields] OR Measure*[All Fields] OR Condition*[All Fields] OR Principle*[All Fields] OR Requirement*[All Fields] OR Scale*[All Fields] OR Parameter*[All Fields] OR Indicator*[All Fields] OR Norm*[All Fields]) OR (Defin*[All Fields] OR Mean*[All Fields] OR Description [All Fields] OR Character*[All Fields] OR Explan*[All Fields] OR delineate [All Fields] OR detail [All Fields] OR interpret[All Fields] OR determine[All Fields] OR elucidate[All Fields] OR illustrate[All Fields] OR exemplify[All Fields])) AND ("Rare Diseases"[Mesh] OR "Orphan disease*"[All Fields] OR "Rare condition*"[All Fields] OR "Rare disorder*"[All Fields] OR "Rare disability*"[All Fields] OR "Neglected disease*"[All Fields] OR "Undiagnosed disease*"[All Fields] OR "Low-frequency disease*"[All Fields] OR "life-threatening disease*"[All Fields] OR "debilitating disease*"[All Fields] OR "severe disease*"[All Fields] OR "intractable disease*"[All Fields])) AND ("Orphan Drug Production"[Mesh] OR "Orphan medicinal product*" [All Fields] OR "Orphan product*"[All Fields] OR "Orphan subset*"[All Fields] OR "Orphan indication*"[All Fields] OR "Highly specialized technolog*"[All Fields] OR "Priority review drug*"[All Fields] OR "Orphan Drug*"[All Fields])	1 OR 2 And 3 and 4	1 OR 2 And 3 and 4	(TITLE-ABS-KEY (criteria OR standard* OR classification OR measure* OR condition* OR principle* OR requirement* OR scale* OR parameter* OR indicator* OR norm*)) OR (TITLE-ABS-KEY (defin* OR mean* OR description OR character* OR explan* OR delineate OR detail OR interpret OR determine OR elucidate OR illustrate OR exemplify)) AND (TITLE-ABS-KEY ("Orphan disease*" OR "Rare condition*" OR "Rare disorder*" OR "Rare disability*" OR "Neglected disease*" OR "Undiagnosed disease*" OR "Low-frequency disease*" OR "life-threatening disease*" OR "debilitating disease*" OR "severe disease*" OR "intractable disease*" OR "Rare Disease*")) AND (TITLE-ABS-KEY ("Orphan medicinal product*" OR "Orphan product*" OR "Orphan subset*" OR "Orphan indication*" OR "Highly specialized technolog*" OR "Priority review drug*" OR "Orphan Drug Production*"))	#7 AND #6 AND #5 Timespan: All years. Indexes: SCI-EXPANDED, SSCI, A&HCI, CPCI-S, CPCI-SSH, ESCI. ...Less

Abozaid GM, *et al. BMJ Open* 2022; 12:e062126. doi: 10.1136/bmjopen-2022-062126
