# Supplementary material for: Criteria to define rare diseases and orphan drugs: a systematic review protocol
Source: BMJ Open. 2022 Jul 29;12(7):e062126. doi: 10.1136/bmjopen-2022-062126 (PMC9345065; doi:10.1136/bmjopen-2022-062126)
Supplement: Author's manuscript [file bmjopen-2022-062126.draft_revisions.pdf]

BMJ Open is committed to open peer review. As part of this commitment we make the peer review history of every article we publish publicly available.

When an article is published we post the peer reviewers' comments and the authors' responses online. We also post the versions of the paper that were used during peer review. These are the versions that the peer review comments apply to.

The versions of the paper that follow are the versions that were submitted during the peer review process. They are not the versions of record or the final published versions. They should not be cited or distributed as the published version of this manuscript.

BMJ Open is an open access journal and the full, final, typeset and author-corrected version of record of the manuscript is available on our site with no access controls, subscription charges or pay-per-view fees (http://bmjopen.bmj.com).

If you have any questions on BMJ Open's open peer review process please email info.bmjopen@bmj.com

BMJ Open

Criteria to Define Rare Diseases and Orphan Drugs: A Systematic Review Protocol

Journal:	*BMJ Open*
Manuscript ID	bmjopen-2022-062126
Article Type:	Protocol
Date Submitted by the Author:	30-Mar-2022
Complete List of Authors:	Abozaid, Ghada; Princess Nourah bint Abdulrahman University, Pharmacy Practice Department; Queen's University Belfast, Centre of Public Health<br>Kerr, Katie; Queen's University Belfast, Centre of Public Health<br>McKnight, Amy; Queen's University Belfast, Centre for Public Health<br>Al-Omar, Hussain A.; King Saud University; Ministry of Health
Keywords:	EPIDEMIOLOGY, HEALTH ECONOMICS, Health policy < HEALTH SERVICES ADMINISTRATION & MANAGEMENT

SCHOLARONE™
Manuscripts

Criteria to Define Rare Diseases and Orphan Drugs: A Systematic Review Protocol

Ghada Mohammed Abozaid[1,2], Katie Kerr[2], Amy Jayne McKnight[2], Hussain Abdulrahman Al-Omar[3,4,5*]

[1]Pharmacy Practice Department, College of Pharmacy, Princess Nourah bint Abdulrahman University, Riyadh, Saudi Arabia. Email: gaabozeed@pnu.edu.sa , ORCiD: https://orcid.org/0000-0002-4523-0577

[2]Centre for Public Health, Institute of Clinical Sciences B, Royal Victoria Hospital, Queen's University Belfast School of Medicine, Dentistry and Biomedical Sciences, Belfast, UK. Email:gabozaid01@qub.ac.uk, ORCiD: https://orcid.org/0000-0002-4523-0577; Email: katie.kerr@qub.ac.uk, ORCiD: https://orcid.org/0000-0002-8469-8885; Email: a.j.mcknight@qub.ac.uk, ORCiD: https://orcid.org/0000-0002-7482-709X

[3]Department of Clinical Pharmacy, College of Pharmacy, King Saud University, P.O. Box: 2457, Riyadh , Zip Code: 11451, Saudi Arabia.
Email: halomar@ksu.edu.sa, ORCiD: https://orcid.org/0000-0002-0765-0466

[4]Health Technology Assessment Unit, College of Pharmacy, King Saud University, P.O. Box: 2457, Riyadh, Zip Code: 11451, Saudi Arabia.

[5]Center of Health Technology Assessment, Ministry of Health, Riyadh, Saudi Arabia.

***Corresponding author:**

Hussain Abdulrahman Al-Omar. MSc, PhD.
Associate Professor of Pharmacoeconomics and Pharmaceutical Policy
Department of Clinical Pharmacy
College of Pharmacy
King Saud University
P.O. Box: 2457, Riyadh
Zip Code: 11451, Saudi Arabia
Email: halomar@ksu.edu.sa
Telephone:0114673637

Word count: 2482.

For peer review only - http://bmjopen.bmj.com/site/about/guidelines.xhtml

Keywords: Orphan Drugs; Rare Diseases; Systematic Review; Criteria; Definition.

Abstract

Introduction

Rare diseases (RDs) are chronic, and life-threatening medical conditions affecting a low percentage of the population compared to other diseases. These conditions are treated with a medications known as orphan drugs (ODs). Unfortunately, there is no single universal definition for RDs or ODs. This systematic review (SR) will identify the quantitative and qualitative criteria used to define RDs, and ODs and the underlying rationale, internationally.

Methods and analysis

This protocol follows the preferred reporting items for systematic review and meta-analysis protocols. A SR will be conducted, including a search of the following databases: PubMed, Medline, EMBASE, Scopus, Web of Science, GreyLit and OpenGrey. Eligible publications will be selected beased on inclusion criteria. Extracted data will be analysed using thematic and content analyses for qualitative descriptors, while quantitative data will be analysed descriptively and reported in the form of frequencies and percentages.

Ethics and dissemination

No ethical approval is required since this SR is focused on secondary analysis of data retrieved from scientific literature. The outcomes of this SR will be published as a part of a PhD theiss, presented at conferences, and published in peer-reviewed journals.

PROSPERO registration number ID CRD42021252701.

Strengths and limitations

- This research describes a comprehensive SR of literature that has been published and has reported qualitative and/or quantitative criteria used to define RDs and ODs.
- It will summarise definitions and criteria literate in relation to studies' designs, geographical locations, methodological rigour and primary outcomes.
- It will act as a foundation in proposing a national definition for RD and OD in Saudi Arabia.

For peer review only - http://bmjopen.bmj.com/site/about/guidelines.xhtml

- Current published definitions depend mainly on disease prevalence. These definitions may over- or underestimate the actual prevalence of RDs due to increasing population sizes and improved identification or recording of RDs.
- The SR will exclude RDs caused by infections, toxins, or cancer.

Introduction

A rare disease (RD) is a health condition with low prevalence compared with common diseases[1]. RDs affect approximately 6% of the worldwide population[2], which equates to >470 million persons. Many RD patients experience difficulties accessing appropriate treatment options. Worldwide, less than one-tenth of patients with RDs have received disease-specific treatment[3]. The varied terminology and inconsistent definitions for RDs are considered a major challenge in treatment accessibility. Unfortunately, there is no single, unified and universally accepted definition for RD [4].

Several international definitions for RD have been proposed and used based on stakeholders' priorities and perspectives. Stakeholders have included scientific societies, patients' groups, regulatory agencies, industry, reimbursement agencies, payers, and decision- and policy-makers. For instance, payers look at RD from health care spending and resource utilization views, which is different from the patient groups' perspectives since their primary goal is often focused on treatment accessibility, as opposed to that of policy-makers, whose priority is improving the efficiency of health care delivery and the health care system. Therefore, it is important to understand the context and application of RD definitions used by different stakeholders[4].

The reported criteria used to define RD differ between countries and organisations. They may be based on either qualitative (subjective), or quantitative (objective, measurable) descriptors. A few definitions use qualitative norms allied with emotional connections, such as disease severity, being life-threatening, inheritable, and if there are alternative treatments[4] [5]. These subjective descriptors are not supported by substantive evidence; they differ depending on the type of organisation that is using the term. For example, the European Organization for Rare Diseases (EURORDIS) incorporates both RDs and neglected diseases under the category of "orphan diseases"[6]. Quantitative descriptors, such as disease prevalence threshold, was the

For peer review only - http://bmjopen.bmj.com/site/about/guidelines.xhtml

preferred epidemiology measured element that was used in 58% of RD definitions based on data from 2014[4], either explicitly or implicitly. Prevalence describes data on the proportion of a particular population living with a disease at a specific time [7] rather than incidence, which reflects the rate of occurrence. Prevalence data can help in determining the needs of RD patients at a population level by conducting economic evaluations, estimating financial recourses required for health and social services, estimating diseases burdens, and offering optimal designs for clinical trials[2]. However, a prevalence threshold is challenging as circumstances change in specific countries; for example, some diseases might be considered as RDs in one country but not in another. In this case, the prevalence of RDs will increase as their identification and treatment with improved survival progresses[2].

In the United Kingdom (UK), the 2021 Rare Disease framework defined RD based on prevalence as a condition affecting fewer than 1 in 2,000 people[8]. Meanwhile, the European Union (EU) countries use the qualitative and quantitative criteria to define RDs as being *"life-threatening or chronically debilitating diseases which are of such low prevalence (less than 5 per 10,000) that special combined efforts are needed to address them so as to prevent significant morbidity or perinatal or early mortality or a considerable reduction in an individual's quality of life or socio-economic potential"*[9]. Worldwide, different organisations have established their own definitions for RDs; for instance, the Food and Drug Administration (FDA) in the United States of America (USA) define an RD as *"any disease or condition that affects less than 200,000 people in the United States or affects >200,000 in the United States and for which there is no reasonable expectation that the cost of developing and making available in the United States a drug for such disease or condition will be recovered from sales in the United States of such drug".* [4] The criterion used in Australia and Japan assumes that a rare disease is one that affects 11 and 40 people out of 100,000 people, respectively[10]. Although these are widely used definitions, some countries adopt others. Argentina, for instance, defines RD using the EU definition [11], while Brazil fosters the World Health Organization definition of *"those affecting less than 65 out of 100,000 individuals"* [12].

As RDs individually affect a small number of people, they are "orphaned" by the pharmaceutical industry, which has promoted the use of the term "orphan disease" when referring to rare

For peer review only - http://bmjopen.bmj.com/site/about/guidelines.xhtml

conditions [13]. To deal with this therapeutic gap in orphan diseases, several countries have established mechanisms to encourage pharmaceutical companies to invest and manufacture orphan drugs (ODs).[3 10 11 14.]

OD is a designation status issued by regulatory bodies to describe therapies that treat rare conditions[15 16]. The pharmaceutical companies that apply for such a designation are incentivized by these regulatory bodies if their therapy fulfils the designation criteria [15 16] OD definitions vary beyond national boundaries. The UK [17], for instance, shares the same European Medicines Agency criteria, which is " *if the drug is intended for the diagnosis, prevention or treatment of a life-threatening or chronically and seriously debilitating condition affecting not more than 5 in 10,000 EU people or that it is unlikely that marketing the drug in the EU would generate sufficient benefit for the affected people and for the drug manufacturer to justify the investment"*[14]. Meanwhile, the FDA define an OD as "*one intended for the treatment, prevention or diagnosis of a rare disease or condition, which is one that affects less than 200,000 persons in the US*" (which equates to approximately six cases per 10,000 population) *"or meets cost recovery provisions of the act*"[18 19]. Both definitions depend on prevalence as a criterion for the designation. In contrast, other countries may have more than one criterion, including prevalence, to designate an OD. For instance, in Japan, to designate a drug as orphan, the drug must meet one of three criteria; the drug is used to treat an RD; there are no other treatments available in Japan; or the proposed drug is clinically superior to drugs already available on the Japanese market[14]. According to section 16H of the Therapeutic Goods Regulations 1997 in Australia, ODs are defined as "*a medicine, vaccine, or in vivo diagnostic agent consider an orphan drug if it intended to treat, prevent or diagnose a rare disease; or must not be commercially viable to supply to treat, prevent, or diagnose another disease or condition*"[14 20.]

As already mentioned, some countries foster an international RD definition by using other country's criteria, which can be problematic due to differences in regional demographic, governmental, economic, and socio-cultural considerations[4]. For instance, consanguineous marriage (CM) is a major risk factor for congenital abnormalities and genetic diseases that are inherited in an autosomal recessive manner, and which result in some RDs[21]. In Saudi Arabia, there is no national definition for RD or OD, even though CM represents 70% of the total

For peer review only - http://bmjopen.bmj.com/site/about/guidelines.xhtml

marriages in the country.[22 21 23 24] Therefore, it is improper for Saudi to espouse or adopt other countries' definitions.

This SR protocol describes the approach which will be utilised to review the published literature on criteria used to define RDs and ODs from both qualitative and quantitative points of view and explores the rationale behind each criterion.

Review aims and objectives

Aim

This study aims to perform a systematic literature review to identify criteria used to define RDs and ODs from both qualitative and quantitative points of view.

Objectives

1. To identify the quantitative criteria used to define RDs and ODs
2. To identify the qualitative criteria used to define RDs and ODs
3. To explore the rationale behind the quantitative criteria used to define RDs and ODs
4. To explore the rationale behind the qualitative criteria used to define RDs and ODs
5. To explore the methodological characteristics that explain any heterogeneity in the results

Methods and analysis

This protocol is registered in PROSPERO (ID CRD42021252701) and will follows the preferred reporting items for systematic review and meta-analysis protocols (PRISMA-P:2015)[25].

Inclusion criteria

The study will include any scientific publication of any design which discuss RD and OD and answers the research question: *What are the criteria to define Rare Diseases & Orphan Drugs globally?* Publications will be eligible for inclusion in the SR based on the PICO framework [population, intervention, comparison, and outcome]. There will be no restriction on publication year, or jurisdiction (North America, South America, Asia, Europe, Africa, Oceania, or the Middle East). We will include studies published in the English language reporting data for the general human population, with data extracted separately for children and adults if reported separately. In this study, the comparator element of the PICO framework will not be

For peer review only - http://bmjopen.bmj.com/site/about/guidelines.xhtml

applicable. Eligible articles will be analysed by looking for a definition for RD, and OD in general. These definitions will be categorised based on qualitative and quantitative criteria as an outcome of the review.

Exclusion criteria

RD due to infection will be excluded as some infections may be rare in wealthy countries while being relatively common in poorer countries: for instance, tuberculosis[26]. Exposure to toxic substances can cause RD and these will also be excluded from this review; for example, asbestos exposure (fibrous silicate mineral) can cause mesothelioma (a type of cancer that develops in the thin layer of tissue that covers the lungs and chest wall).[26]. Furthermore, rare forms of cancers will be excluded in our SR as there are discrepancies in rarity between countries that are typically captured by national cancer registries; hepatocellular carcinoma is common in China (95.7/100 000 people) but is relatively uncommon in Canada (6.8/100 000 people),[27 28.]

Search strategy

A systematic literature review will be conducted using Boolean Operator rules ('AND', 'OR', and 'NOT') with 'truncation' to broaden the search terms with various word spellings and endings. The following search terms (ultra-orphan disease, ultra-orphan drug, ultra-rare disease, orphan disease, orphan drug, orphan medicinal product, orphan product, orphan subset, orphan indication, orphan pharmaceutical product, orphan designation, neglected disease, rare and neglected disease, rare condition, rare disease, rare disorder, rare disability, rare medicinal technology, syndrome without a name, undiagnosed disease, life-threatening, debilitating, severe, and intractable, highly specialized technology, very rare disease, low-frequency disease, pharmacological therapies of high complexity, priority review drugs, extremely rare disease, and orphan drug reimbursement system) were discussed and validated as terms by a library information specialist and research team: see *Appendix (1)*. Publications will be retrieved from different databases, including PubMed, Medline, EMBASE, Scopus, Web of Science (science and social science citation index), and grey literature databases including GreyLit and OpenGrey. To maximise literature saturation, this search will be complemented by reverse citation screening of relevant articles and forward citation searching. Databases will be searched from their commencement until 17th December 2021.

For peer review only - http://bmjopen.bmj.com/site/about/guidelines.xhtml

Study selection

Studies will be selected after publications are compiled and duplicates will be removed. Then, two authors (GA and KK) will conduct two rounds of screening independently for the titles and abstracts for initial eligibility based on the inclusion and exclusion criteria. The full text of the included publications that appear to meet the inclusion criteria, and when there is insufficient information in the title and abstract to exclude the study, will be retrieved. Disagreements between individual reviewers will be resolved by a third reviewer (AJM) , and all decisions will be documented in a Microsoft® Excel® spreadsheet. None of the authors will be blind to the journal titles, nor the study authors or institutions.

Data extraction

The included articles will be summarised using a custom spreadsheet developed in a primary screening, quality assessment, and data extraction web-based software platform tool, Covidence®[29]. The data will be extracted by two authors (GA and KK) independently. For missing, unreported data or additional details, the study's primary investigator (GA) will attempt to contact the study authors. The timeframe for a reply before the article is excluded, based on the requested information, is three weeks.

Extracted data will include author(s) name(s), publication year, publication type, journal title, study design, organisation, geographical location, population, results of a study quality assessment, and definitions of RD and OD. Moreover, the types of RD and OD criteria (qualitative or quantitative), their descriptor criteria, the rationale behind the definition, the status of the definition (developed or adopted), and if RD and OD definitions have been considered (implicit or explicit) will also be recorded.

Critical appraisal of studies

Data extraction will be performed, and methodological rigour critically appraised by two authors (GA and KK) separately using customised forms based on the Joanna Briggs Institute (JBI) checklists[30]. JBI's critical appraisal tools assist in assessing the trustworthiness, relevance, and the results of published studies. Moreover, grey literature will be evaluated and critically

For peer review only - http://bmjopen.bmj.com/site/about/guidelines.xhtml

appraised using an AACODS checklist (authority, accuracy, coverage, objectivity, date, significance)[31].

Data synthesis

Narrative synthesis from multiple studies will be undertaken to summarise and explain the current state of knowledge in relation to the SR's aim and objectives. The initial description of the included study's results will be developed using a preliminary synthesis in the form of thematic and content analyses for qualitative descriptors. Descriptive analysis will be conducted in the form of frequencies and percentages for quantitative descriptors, the results being presented in a tabular format.

These data will be processed by different quantitative statistical and qualitative analysis approaches to investigate the similarities and differences between the studies and to explore the relationships within the data, aiming to report key elements for defining RDs and ODs qualitatively and quantitatively. Risk of bias will be assessed considering diversity in study designs, populations, and outcomes.

Patient and Public Involvement

During the protocol development there were no public or patient involvement. While, the SR is focused on a secondary analysis of literature in the public domain, So they will be none in the SR.

Ethics and dissemination

Ethical committee approval is not required to conduct this SR as no primary data will be collected; this SR is focused on a secondary analysis of literature in the public domain. It is part of a PhD project at Queen's University Belfast and the results will be presented at conferences, published as a thesis, and we anticipate publication in a peer-reviewed journal. The results will guide stakeholders to define RDs and ODs and will inform policy decisions and future research.

Acknowledgement

The authors would like to thank Richard Fallis, library information specialist, for his assistance in verifying the search terms and strategy in this study.

For peer review only - http://bmjopen.bmj.com/site/about/guidelines.xhtml

Author contributions:

AJM & HA are responsible for the research supervision, while GA, AJM & HA have conceived, conceptualised and designed this study. GA, KK, AJM and HA have contributed to data acquisition and analyses of the data. All the authors have contributed to the interpretation of the results. GA initially drafted this manuscript, then all the authors reviewed the manuscript for important intellectual content, approved the final version submitted for publication, and agreed to be accountable for all aspects of the research.

Funding

This SR is supported by the Medical Research Council Northern Ireland Executive in support of the Northern Ireland Genomic Medicine Centre through the Belfast Health and Social Care Trust award number (MC_PC_16018), and a Science Foundation Ireland and Department for the Economy, Northern Ireland partnership award number (15/IA/3152). Princess Nourah University, Riyadh, Saudi Arabia support this study through PhD scholarship with no grant number. These funders have no role in developing this protocol.

Competing interests

The authors have no conflict of interests to declare.

Data sharing statement

GA, KK, and AJMconfirm that the data providng the findings of the SR will be available within the article and its supplementary materials (study protocol and statisitcel analysis plan).

For peer review only

For peer review only - http://bmjopen.bmj.com/site/about/guidelines.xhtml

References

1. Gorini F, Coi A, Mezzasalma L, et al. Survival of patients with rare diseases: a population-based study in Tuscany (Italy). *Orphanet J Rare Dis* 2021;16(1):275. doi: 10.1186/s13023-021-01907-0 [published Online First: 20210614]
2. Nguengang Wakap S, Lambert DM, Olry A, et al. Estimating cumulative point prevalence of rare diseases: analysis of the Orphanet database. *Eur J Hum Genet* 2020;28(2):165-73. doi: 10.1038/s41431-019-0508-0 [published Online First: 20190916]
3. Chan AYL, Chan VKY, Olsson S, et al. Access and Unmet Needs of Orphan Drugs in 194 Countries and 6 Areas: A Global Policy Review With Content Analysis. *Value Health* 2020;23(12):1580-91. doi: 10.1016/j.jval.2020.06.020 [published Online First: 20201031]
4. Richter T, Nestler-Parr S, Babela R, et al. Rare Disease Terminology and Definitions-A Systematic Global Review: Report of the ISPOR Rare Disease Special Interest Group. *Value Health* 2015;18(6):906-14. doi: 10.1016/j.jval.2015.05.008 [published Online First: 20150818]
5. Steven Simoens DC, Marc Dooms, Eline Picavet. Orphan Drugs for Rare Diseases. *Drugs* 2012(72):1437–43 doi: https://doi.org/10.2165/11635320-000000000-00000
6. Rode J. Rare Diseases: understanding this Public Health Priority: Eurordis; 2005 [Available from: https://www.eurordis.org/IMG/pdf/princeps_document-EN.pdf2021.
7. Melissa Conrad Stöppler M. Medical Definition of Prevalence 2021 [Available from: https://www.medicinenet.com/prevalence/definition.htm.
8. Lord B. RS, Vaughan G., Mairi G. The UK Rare Diseases Framework 2021 [Available from: https://www.gov.uk/government/publications/uk-rare-diseases-framework/the-uk-rare-diseases-framework.
9. Regulation (EC) No 141/2000 of the European Parliament and of the Council of 16 December 1999 on orphan medicinal products. 1999 [Available from: http://eur-lex.europa.eu/smartapi/cgi/sga_doc?smartapi!celexapi!prod!CELEXnumdoc&lg=en&numdoc=32000R0141&model=guichett).
10. Stawowczyk E, Malinowski KP, Kawalec P, et al. Reimbursement Status and Recommendations Related to Orphan Drugs in European Countries. *Front Pharmacol* 2019;10:1279. doi: 10.3389/fphar.2019.01279 [published Online First: 20191127]

For peer review only - http://bmjopen.bmj.com/site/about/guidelines.xhtml

11. Arnold RJ, Bighash L, Bryon Nieto A, et al. The role of globalization in drug development and access to orphan drugs: orphan drug legislation in the US/EU and in Latin America. *F1000Res* 2015;4:57. doi: 10.12688/f1000research.4268.1 [published Online First: 20150227]

12. Passos-Bueno MR, Bertola D, Horovitz DD, et al. Genetics and genomics in Brazil: a promising future. *Mol Genet Genomic Med* 2014;2(4):280-91. doi: 10.1002/mgg3.95 [published Online First: 2014/08/01]

13. Rare and Orphan Lung Disease European Lung Foundation2021 [Available from: https://europeanlung.org/en/information-hub/lung-conditions/rare-and-orphan-lung-disease/.

14. Sharma A, Jacob A, Tandon M, et al. Orphan drug: Development trends and strategies. *J Pharm Bioallied Sci* 2010;2(4):290-9. doi: 10.4103/0975-7406.72128 [published Online First: 2010/12/25]

15. European Medicines Agency. Orphan designation 2022 [Available from: https://www.ema.europa.eu/en/glossary/orphan-designation accessed 06/01/2022.

16. U.S. Food and Drugs Administration. Drug Designation 12/17/2021 [Available from: https://www.fda.gov/animal-veterinary/minor-useminor-species/drug-designation accessed 06/01/2022.

17. Agency MaHpR. Guidance Orphan medicinal products 31 December 2020 [Available from: https://www.gov.uk/guidance/orphan-medicinal-products-in-great-britain.

18. Developing Products for Rare Diseases & Conditions US Food and Drug Adminstration20 December 2018. Retrieved 28 December 2019 [Available from: https://www.fda.gov/industry/developing-products-rare-diseases-conditions.

19. Hall AK, Carlson MR. The current status of orphan drug development in Europe and the US. *Intractable Rare Dis Res* 2014;3(1):1-7. doi: 10.5582/irdr.3.1 [published Online First: 2014/10/25]

20. Herkes GK. Orphan drugs in Australia. *Expert Opinion on Orphan Drugs* 2016;4(12):1195-97. doi: 10.1080/21678707.2016.1257383

21. Alahdal H, Alshanbari H, Almazroa H, et al. Consanguinity, awareness, and genetic disorders among female university students in Riyadh, Saudi Arabia. *Journal of Biochemical and Clinical Genetics* 2021;4(1):27-34. doi: 10.24911/JBCGenetics/183-1601264923

For peer review only - http://bmjopen.bmj.com/site/about/guidelines.xhtml

22. Al-Gazali L, Hamamy H, Al-Arrayad S. Genetic disorders in the Arab world. *BMJ* 2006;333(7573):831-4. doi: 10.1136/bmj.38982.704931.AE

23. AbdulAzeez S, Al Qahtani NH, Almandil NB, et al. Genetic disorder prenatal diagnosis and pregnancy termination practices among high consanguinity population, Saudi Arabia. *Sci Rep* 2019;9(1):17248. doi: 10.1038/s41598-019-53655-8 [published Online First: 20191121]

24. Mahboub SM, Alsaqabi AA, Allwimi NA, et al. Prevalence and pattern of consanguineous marriage among educated married individuals in Riyadh. *J Biosoc Sci* 2020;52(5):768-75. doi: 10.1017/S0021932019000786 [published Online First: 20191129]

25. Moher D, Shamseer L, Clarke M, et al. Preferred reporting items for systematic review and meta-analysis protocols (PRISMA-P) 2015 statement. *Syst Rev* 2015;4:1. doi: 10.1186/2046-4053-4-1 [published Online First: 20150101]

26. Development IoMUCoARDRaOP. Rare Diseases and Orphan Products: Accelerating Research and Development. Washington (DC): National Academies Press (US), 2010.

27. McGlynn KA, London WT. Epidemiology and natural history of hepatocellular carcinoma. *Best Pract Res Clin Gastroenterol* 2005;19(1):3-23. doi: 10.1016/j.bpg.2004.10.004 [published Online First: 2005/03/11]

28. De P, Dryer D, Otterstatter MC, et al. Canadian trends in liver cancer: a brief clinical and epidemiologic overview. *Curr Oncol* 2013;20(1):e40-3. doi: 10.3747/co.20.1190 [published Online First: 2013/02/28]

29. Innovation VH. Covidence systematic review software Melbourne, AustraliaFebruary 4, 2019 [Available from: https://www.covidence.org/2021.

30. Institute JB. Critical appraisal tools 2020 [Available from: https://jbi.global/critical-appraisal-tools.

31. Tyndall J. AACODS Checklist. 2010 [Available from: https://dspace.flinders.edu.au/xmlui/bitstream/handle/2328/3326/AACODS_Checklist.pdf?sequence=4&isAllowed=y.

For peer review only - http://bmjopen.bmj.com/site/about/guidelines.xhtml

Appendix 1: Search strategy

Research question: What are the criteria to define Rare Diseases and Orphan Drugs globally?

	PubMed	Medline	Embase	Scopus	WOS
Concept 1 (Criteria)	Criteria [All Fields] OR Standard*[All Fields] OR classification [All Fields] OR Measure*[All Fields] OR Condition*[All Fields] OR Principle*[All Fields] OR Requirement*[All Fields] OR Scale*[All Fields] OR Parameter*[All Fields] OR Indicator*[All Fields] OR Norm*[All Fields]	(Criteria or Standard* or classification or Measure* or Condition* or Principle* or Requirement* or Scale* or Parameter* or Indicator* or Norm*).mp. [mp=title, abstract, original title, name of substance word, subject heading word, floating sub-heading word, keyword heading word, organism supplementary concept word, protocol supplementary concept word, rare disease supplementary concept word, unique identifier, synonyms]	(Criteria or Standard* or classification or Measure* or Condition* or Principle* or Requirement* or Scale* or Parameter* or Indicator* or Norm*).mp. [mp=title, abstract, heading word, drug trade name, original title, device manufacturer, drug manufacturer, device trade name, keyword, floating subheading word, candidate term word]	TITLE-ABS-KEY (criteria OR standard* OR classification OR measure* OR condition* OR principle* OR requirement* OR scale* OR parameter* OR indicator* OR norm*)	ALL FIELDS: (criteria OR standard* OR classification OR measure* OR condition* OR principle* OR requirement* OR scale* OR parameter* OR indicator* OR norm*) Timespan: All years. Indexes: SCI-EXPANDED, SSCI, A&HCI, CPCI-S, CPCI-SSH, ESCI.
Concept 2 (Define)	Defin*[All Fields] OR Mean*[All Fields] OR Description [All Fields] OR Character*[All Fields] OR Explan*[All Fields] OR delineate [All Fields] OR detail [All Fields] OR interpret[All Fields] OR determine[All Fields] OR elucidate[All Fields] OR illustrate[All Fields] OR exemplify[All Fields]	(Defin* or Mean* or Description or Character* or Explan* or delineate or detail or interpret or determine or elucidate or illustrate or exemplify).mp. [mp=title, abstract, original title, name of substance word, subject heading word, floating sub-heading word, keyword heading word, organism supplementary concept word, protocol supplementary concept word, rare disease supplementary concept word, unique identifier, synonyms]	(Defin* or Mean* or Description or Character* or Explan* or delineate or detail or interpret or determine or elucidate or illustrate or exemplify).mp. [mp=title, abstract, heading word, drug trade name, original title, device manufacturer, drug manufacturer, device trade name, keyword, floating subheading word, candidate term word]	TITLE-ABS-KEY (defin* OR mean* OR description OR character* OR explan* OR delineate OR detail OR interpret OR determine OR elucidate OR illustrate OR exemplify)	ALL FIELDS: (defin* OR mean* OR description OR character* OR explan* OR delineate OR detail OR interpret OR determine OR elucidate OR illustrate OR exemplify) Timespan: All years. Indexes: SCI-EXPANDED, SSCI, A&HCI, CPCI-S, CPCI-SSH, ESCI.
Concept 3 (Rare Disease(s)	"Rare Diseases"[Mesh] OR “Orphan disease*”[All Fields] OR “Rare condition*”[All Fields] OR “Rare disorder*”[All Fields] OR “Rare disability*”[All Fields] OR “Neglected disease*”[All Fields] OR “Undiagnosed disease*”[All Fields] OR “Low-frequency disease*”[All Fields] OR “life-threatening disease*”[All Fields] OR “debilitating disease*”[All Fields] OR “severe disease*”[All Fields] OR “intractable disease*”[All Fields]	(Orphan disease* or Rare condition* or Rare disorder* or Rare disability* or Neglected disease* or Undiagnosed disease* or Low-frequency disease* or life-threatening disease* or debilitating disease* or severe disease* or intractable disease* or Rare Disease*).mp. [mp=title, abstract, original title, name of substance word, subject heading word, floating sub-heading word, keyword heading word, organism supplementary concept word, protocol supplementary concept word, rare disease supplementary concept word, unique identifier, synonyms]	(Orphan disease* or Rare condition* or Rare disorder* or Rare disability* or Neglected disease* or Undiagnosed disease* or Low-frequency disease* or life-threatening disease* or debilitating disease* or severe disease* or intractable disease* or Rare Disease*).mp. [mp=title, abstract, heading word, drug trade name, original title, device manufacturer, drug manufacturer, device trade name, keyword, floating subheading word, candidate term word]	TITLE-ABS-KEY ("Orphan disease*" OR "Rare condition*" OR "Rare disorder*" OR "Rare disability*" OR "Neglected disease*" OR "Undiagnosed disease*" OR "Low-frequency disease*" OR "life-threatening disease*" OR "debilitating disease*" OR "severe disease*" OR "intractable disease*" OR "Rare Disease*")	ALL FIELDS: ("Orphan disease*" OR “Rare condition*” OR “Rare disorder*” OR “Rare disability*” OR “Neglected disease*” OR “Undiagnosed disease*” OR “Low-frequency disease*” OR “life-threatening disease*” OR “debilitating disease*” OR “severe disease*” OR “intractable disease*” OR “Rare Disease*”) Timespan: All years. Indexes: SCI-EXPANDED, SSCI, A&HCI, CPCI-S, CPCI-SSH, ESCI.

For peer review only - http://bmjopen.bmj.com/site/about/guidelines.xhtml

Concept 4 (Orphan Drug(s))	"Orphan Drug Production"[Mesh] OR "Orphan medicinal product*" [All Fields] OR "Orphan product*"[All Fields] OR "Orphan subset*"[All Fields] OR "Orphan indication*"[All Fields] OR "Highly specialized technolog*"[All Fields] OR "Priority review drug*"[All Fields] OR "Orphan Drug*"[All Fields]	(Orphan medicinal product* or Orphan product* or Orphan subset* or Orphan indication* or Highly specialized technolog* or Priority review drug* or Orphan Drug* or Orphan Drug Production*).mp. [mp=title, abstract, original title, name of substance word, subject heading word, floating sub-heading word, keyword heading word, organism supplementary concept word, protocol supplementary concept word, rare disease supplementary concept word, unique identifier, synonyms]	(Orphan medicinal product* or Orphan product* or Orphan subset* or Orphan indication* or Highly specialized technolog* or Priority review drug* or Orphan Drug* or Orphan Drug Production*).mp. [mp=title, abstract, heading word, drug trade name, original title, device manufacturer, drug manufacturer, device trade name, keyword, floating subheading word, candidate term word]	TITLE-ABS-KEY ("Orphan medicinal product*" OR "Orphan product*" OR "Orphan subset*" OR "Orphan indication*" OR "Highly specialized technolog*" OR "Priority review drug*" OR "Orphan Drug Production*" OR "Orphan Drug*")	ALL FIELDS: ("Orphan medicinal product*" OR "Orphan product*" OR "Orphan subset*" OR "Orphan indication*" OR "Highly specialized technolog*" OR "Priority review drug*" OR "Orphan Drug Production*" OR "Orphan Drug*") Timespan: All years. Indexes: SCI-EXPANDED, SSCI, A&HCI, CPCI-S, CPCI-SSH, ESCI.
Total	(((Criteria [All Fields] OR Standard*[All Fields] OR classification [All Fields] OR Measure*[All Fields] OR Condition*[All Fields] OR Principle*[All Fields] OR Requirement*[All Fields] OR Scale*[All Fields] OR Parameter*[All Fields] OR Indicator*[All Fields] OR Norm*[All Fields]) OR (Defin*[All Fields] OR Mean*[All Fields] OR Description [All Fields] OR Character*[All Fields] OR Explan*[All Fields] OR delineate [All Fields] OR detail [All Fields] OR interpret[All Fields] OR determine[All Fields] OR elucidate[All Fields] OR illustrate[All Fields] OR exemplify[All Fields])) AND ("Rare Diseases"[Mesh] OR "Orphan disease*"[All Fields] OR "Rare condition*"[All Fields] OR "Rare disorder*"[All Fields] OR "Rare disability*"[All Fields] OR "Neglected disease*"[All Fields] OR "Undiagnosed disease*"[All Fields] OR "Low-frequency disease*"[All Fields] OR "life-threatening disease*"[All Fields] OR "debilitating disease*"[All Fields] OR "severe disease*"[All Fields] OR "intractable disease*"[All Fields])) AND ("Orphan Drug Production"[Mesh] OR "Orphan medicinal product*" [All Fields] OR "Orphan product*"[All Fields] OR "Orphan subset*"[All Fields] OR "Orphan indication*"[All Fields] OR "Highly specialized technolog*"[All Fields] OR "Priority review drug*"[All Fields] OR "Orphan Drug*"[All Fields])	1 OR 2 And 3 and 4	1 OR 2 And 3 and 4	(TITLE-ABS-KEY (criteria OR standard* OR classification OR measure* OR condition* OR principle* OR requirement* OR scale* OR parameter* OR indicator* OR norm*)) OR (TITLE-ABS-KEY (defin* OR mean* OR description OR character* OR explan* OR delineate OR detail OR interpret OR determine OR elucidate OR illustrate OR exemplify)) AND (TITLE-ABS-KEY ("Orphan disease*" OR "Rare condition*" OR "Rare disorder*" OR "Rare disability*" OR "Neglected disease*" OR "Undiagnosed disease*" OR "Low-frequency disease*" OR "life-threatening disease*" OR "debilitating disease*" OR "severe disease*" OR "intractable disease*" OR "Rare Disease*")) AND (TITLE-ABS-KEY ("Orphan medicinal product*" OR "Orphan product*" OR "Orphan subset*" OR "Orphan indication*" OR "Highly specialized technolog*" OR "Priority review drug*" OR "Orphan Drug Production*"))	#7 AND #6 AND #5 Timespan: All years. Indexes: SCI-EXPANDED, SSCI, A&HCI, CPCI-S, CPCI-SSH, ESCI. ...Less

For peer review only - http://bmjopen.bmj.com/site/about/guidelines.xhtml

PRISMA-P 2015 Checklist

This checklist has been adapted for use with protocol submissions to *Systematic Reviews* from Table 3 in Moher D et al: Preferred reporting items for systematic review and meta-analysis protocols (PRISMA-P) 2015 statement. *Systematic Reviews* 2015 **4**:1

Section/topic	#	Checklist item	Information reported Yes	Information reported No	Line number(s)
**ADMINISTRATIVE INFORMATION**					
**Title**					
Identification	1a	Identify the report as a protocol of a systematic review	☒	☐	3
Update	1b	If the protocol is for an update of a previous systematic review, identify as such	☐	☒	n/a
**Registration**	2	If registered, provide the name of the registry (e.g., PROSPERO) and registration number in the Abstract	☒	☐	61, 174
**Authors**					
Contact	3a	Provide name, institutional affiliation, and e-mail address of all protocol authors; provide physical mailing address of corresponding author	☒	☐	4-22
Contributions	3b	Describe contributions of protocol authors and identify the guarantor of the review	☒	☐	338-344
**Amendments**	4	If the protocol represents an amendment of a previously completed or published protocol, identify as such and list changes; otherwise, state plan for documenting important protocol amendments	☐	☒	n/a
**Support**					
Sources	5a	Indicate sources of financial or other support for the review	☒	☐	346-350
Sponsor	5b	Provide name for the review funder and/or sponsor	☒	☐	346-350
Role of sponsor/funder	5c	Describe roles of funder(s), sponsor(s), and/or institution(s), if any, in developing the protocol	☒	☐	350
**INTRODUCTION**					
**Rationale**	6	Describe the rationale for the review in the context of what is already known	☒	☐	151-161
**Objectives**	7	Provide an explicit statement of the question(s) the review will address with reference to participants, interventions, comparators, and outcomes (PICO)	☒	☐	167-172 , 178 - 180
**METHODS**					

For peer review only

Section/topic	#	Checklist item	Information reported Yes	Information reported No	Line number(s)
**Eligibility criteria**	8	Specify the study characteristics (e.g., PICO, study design, setting, time frame) and report characteristics (e.g., years considered, language, publication status) to be used as criteria for eligibility for the review	☒	☐	176-187
**Information sources**	9	Describe all intended information sources (e.g., electronic databases, contact with study authors, trial registers, or other grey literature sources) with planned dates of coverage	☒	☐	199- 214
**Search strategy**	10	Present draft of search strategy to be used for at least one electronic database, including planned limits, such that it could be repeated	☒	☐	361
STUDY RECORDS					
Data management	11a	Describe the mechanism(s) that will be used to manage records and data throughout the review	☒	☐	50, 222, 225
Selection process	11b	State the process that will be used for selecting studies (e.g., two independent reviewers) through each phase of the review (i.e., screening, eligibility, and inclusion in meta-analysis)	☒	☐	217, 218
Data collection process	11c	Describe planned method of extracting data from reports (e.g., piloting forms, done independently, in duplicate), any processes for obtaining and confirming data from investigators	☒	☐	216,227-229
**Data items**	12	List and define all variables for which data will be sought (e.g., PICO items, funding sources), any pre-planned data assumptions and simplifications	☒	☐	230-235
**Outcomes and prioritization**	13	List and define all outcomes for which data will be sought, including prioritization of main and additional outcomes, with rationale	☒	☐	230-235
**Risk of bias in individual studies**	14	Describe anticipated methods for assessing risk of bias of individual studies, including whether this will be done at the outcome or study level, or both; state how this information will be used in data synthesis	☒	☐	253
DATA					
**Synthesis**	15a	Describe criteria under which study data will be quantitatively synthesized	☒	☐	92, 98, 186,187
	15b	If data are appropriate for quantitative synthesis, describe planned summary measures, methods of handling data, and methods of combining data from studies, including any planned exploration of consistency (e.g., I^2, Kendall's tau)	☒	☐	52, 53, 250-253
	15c	Describe any proposed additional analyses (e.g., sensitivity or subgroup analyses, meta-regression)	☐	☒	n/a
	15d	If quantitative synthesis is not appropriate, describe the type of summary planned	☒	☐	186, 247, 250 -253
**Meta-bias(es)**	16	Specify any planned assessment of meta-bias(es) (e.g., publication bias across studies, selective reporting within studies)	☐	☒	n/a

For peer review only

BioMed Central The Open Access Publisher

3

Section/topic	#	Checklist item	Information reported		Line number(s)
			Yes	No	
**Confidence in cumulative evidence**	17	Describe how the strength of the body of evidence will be assessed (e.g., GRADE)	☒	☐	237-242

For peer review only

BioMed Central
The Open Access Publisher

BMJ Open

Criteria to Define Rare Diseases and Orphan Drugs: A Systematic Review Protocol

Journal:	*BMJ Open*
Manuscript ID	bmjopen-2022-062126.R1
Article Type:	Protocol
Date Submitted by the Author:	10-Jul-2022
Complete List of Authors:	Abozaid, Ghada; Princess Nourah bint Abdulrahman University, Pharmacy Practice Department; Queen's University Belfast, Centre of Public Health<br>Kerr, Katie; Queen's University Belfast, Centre of Public Health<br>McKnight, Amy; Queen's University Belfast, Centre for Public Health<br>Al-Omar, Hussain A.; King Saud University; Ministry of Health
**Primary Subject Heading**:	Public health
Secondary Subject Heading:	Health policy, Public health, Health economics
Keywords:	EPIDEMIOLOGY, HEALTH ECONOMICS, Health policy < HEALTH SERVICES ADMINISTRATION & MANAGEMENT

SCHOLARONE™ Manuscripts

Criteria to Define Rare Diseases and Orphan Drugs: A Systematic Review Protocol

Ghada Mohammed Abozaid[1,2], Katie Kerr[2], Amy Jayne McKnight[2], Hussain Abdulrahman Al-Omar[3,4,5]*

[1]Pharmacy Practice Department, College of Pharmacy, Princess Nourah bint Abdulrahman University, Riyadh, Saudi Arabia. Email: gaabozeed@pnu.edu.sa , ORCiD: https://orcid.org/0000-0002-4523-0577

[2]Centre for Public Health, Institute of Clinical Sciences B, Royal Victoria Hospital, Queen's University Belfast School of Medicine, Dentistry and Biomedical Sciences, Belfast, UK. Email:gabozaid01@qub.ac.uk, ORCiD: https://orcid.org/0000-0002-4523-0577; Email: katie.kerr@qub.ac.uk, ORCiD: https://orcid.org/0000-0002-8469-8885; Email: a.j.mcknight@qub.ac.uk, ORCiD: https://orcid.org/0000-0002-7482-709X

[3]Department of Clinical Pharmacy, College of Pharmacy, King Saud University, P.O. Box: 2457, Riyadh, Zip Code: 11451, Saudi Arabia.

Email: halomar@ksu.edu.sa, ORCiD: https://orcid.org/0000-0002-0765-0466

[4]Health Technology Assessment Unit, College of Pharmacy, King Saud University, P.O. Box: 2457, Riyadh, Zip Code: 11451, Saudi Arabia.

[5]Center of Health Technology Assessment, Ministry of Health, Riyadh, Saudi Arabia.

***Corresponding author:**

Hussain Abdulrahman Al-Omar. MSc, PhD.
Associate Professor of Pharmacoeconomics and Pharmaceutical Policy
Department of Clinical Pharmacy
College of Pharmacy
King Saud University
P.O. Box: 2457, Riyadh
Zip Code: 11451, Saudi Arabia
Email: halomar@ksu.edu.sa
Telephone:0114673637

Word count: 2709.

Keywords: Orphan Drugs; Rare Diseases; Systematic Review; Criteria; Definition.

For peer review only

For peer review only - http://bmjopen.bmj.com/site/about/guidelines.xhtml

Abstract

Introduction

Rare diseases (RDs) are often chronic and progressive life-threatening medical conditions that affect a low percentage of the population compared to other diseases. These conditions can be treated with medications known as orphan drugs (ODs). Unfortunately, there is no universal definition of RDs or ODs. This systematic review (SR) will identify the quantitative and qualitative criteria and the underlying rationale used internationally to define RDs and ODs.

Methods and analysis

This protocol follows the conventions for the preferred reporting items for systematic review and meta-analysis protocols (PRISMA-P, 2015). A systematic review will be conducted, including a search of the following databases: PubMed, MEDLINE, EMBASE, Scopus, Web of Science, GreyLit, and OpenGrey. Eligible publications will be selected based on predetermined inclusion criteria. Extracted data will be analysed using thematic and content analyses of qualitative descriptors, whereas quantitative data will be analysed descriptively and reported in the form of frequencies and percentages.

Ethics and dissemination

No ethical approval is required since this systematic review focuses on the secondary analysis of data retrieved from the scientific literature. The outcomes of this systematic review will be published as part of a PhD thesis, presented at conferences, and published in peer-reviewed journals.

PROSPERO registration number ID CRD42021252701.

Strengths and limitations

- This document describes a comprehensive systematic review following the Preferred Reporting Items for Systematic Reviews and Meta-Analyses Protocols (PRISMA-P 2015) guidelines;
- It summarises the definitions and criteria retrieved from the literature in relation to study design, geographical location, methodological rigor, and outcomes;

For peer review only - http://bmjopen.bmj.com/site/about/guidelines.xhtml

For peer review only

- Screening of the articles, data extraction, quality, and risk of bias assessment will be conducted independently by two reviewers, with an independent third reviewer resolving the queries;
- The quality of evidence for all outcomes will be judged using the Joanna Briggs Institute's critical appraisal tools for different articles and an AACODS checklist (authority, accuracy, coverage, objectivity, date, significance) for grey literature;
- Relevant studies published in non-English languages may be missed by this review.

Introduction

A rare disease (RD) is a health condition with a low prevalence compared with common diseases[1]. RDs affect approximately 6% of the worldwide population[2]; when extrapolated to the worldwide population in 2022 (7,950,418,550)[3] that is equivalent to >470 million people. Many patients with RDs experience difficulties accessing appropriate treatment options. Globally, less than one-tenth of patients with RDs receive disease-specific treatment[4]. The varied terminology and inconsistent definitions of RDs are considered major challenges in treatment accessibility. The insufficient data relating to what diseases are considered rare deliberated as an obstacle in understanding of these diseases, defining them, disease coding, correctly diagnosed, and gain pharmaceutical interest. Unfortunately, there is no single, unified, and universally accepted definition of RD[5].

Several international definitions of RD have been proposed and used based on different stakeholders' priorities and perspectives5. Stakeholders include scientific societies, patient groups, regulatory agencies, industry, reimbursement agencies, payers, decision makers, and policymakers. For instance, payers view RDs from healthcare spending and resource utilisation standpoints, which is different from the perspectives of patient groups because their primary goal is often focused on treatment accessibility, as opposed to that of policymakers, whose priority is improving the efficiency of healthcare delivery and the healthcare system. Therefore, it is important to understand the context and application of the definitions for RD used by different stakeholders[5].

The criteria used to define RD also differ between countries and organisations. These may be based on either qualitative (subjective) or quantitative (objective, measurable) descriptors. Some definitions use qualitative norms related to emotional connections, such as disease

For peer review only - http://bmjopen.bmj.com/site/about/guidelines.xhtml

severity, whether it is life-threatening or inheritable, or the availability of alternative treatments[5 6]. These subjective descriptors are not supported by substantive evidence; they differ depending on the type of organisation using the term. For example, the European Organization for Rare Diseases (EURORDIS) incorporates both RDs and neglected diseases under the category of "orphan diseases"[7]. Quantitative descriptors, such as the disease prevalence threshold, are the preferred epidemiology-measured elements used in 58% of RD definitions, either explicitly or implicitly based on data from 2014[5]. Prevalence describes the data on the basis of the proportion of a particular population living with a disease at a specific time[8], while incidence reflects the rate of occurrence. Prevalence data can help determine the needs of RD patients at a population level when conducting economic evaluations, estimating the financial resources required for health and social services, estimating disease burdens, and offering optimal designs for clinical trials[2]. However, determining a prevalence threshold is challenging as it originated from a variety of different information sources. This is heightened by lack of determinedly diagnostic criteria or coding systems to obtain this data[9]. Consequently, some diseases might be considered rare in one country but not in another, due to genetic population diversity, environmental or societal pressures, or survival issues in different regions[10].

In the United Kingdom (UK), the 2021 Rare Disease Framework defined RD based on prevalence, as a condition affecting fewer than 1 in 2,000 people[11]. Meanwhile, European Union (EU) countries use qualitative and quantitative criteria to define RDs as being *"life-threatening or chronically debilitating diseases which are of such low prevalence (less than 5 per 10,000) that special combined efforts are needed to address them so as to prevent significant morbidity or perinatal or early mortality or a considerable reduction in an individual's quality of life or socio-economic potential"*[12]. In the United States (US), the Rare Diseases Act (RDA) of 2002[13], precisely defines RD according to prevalence; *"'rare disease' means any disease or condition that affects less than 200,000 persons in the United States".* Prior to the RDA, the Orphan Drug Act (ODA) of 1983[14] was the federal law which facilitated the development of rare disease drugs, and it defined rare diseases based on qualitative descriptors as follows: "*the term 'rare disease or condition' means any disease or condition which occurs so infrequently in the United States that there is no reasonable expectation that the cost of developing and making available in the United*

For peer review only - http://bmjopen.bmj.com/site/about/guidelines.xhtml

States a drug for such disease or condition will be recovered from sales in the United States of such drug". Meanwhile, the Food and Drug Administration (FDA) used both qualitative and quantitative criterion to define RD as *"any disease or condition that affects less than 200,000 people in the United States or affects >200,000 in the United States and for which there is no reasonable expectation that the cost of developing and making available in the United States a drug for such disease or condition will be recovered from sales in the United States of such drug"*[15, 16]. The criteria used in Australia and Japan assume that a rare disease affects 11 or 40 out of 100,000 people, respectively[17]. Although these definitions are widely used, some countries have adopted others. Argentina, for instance, identifies RD using the EU definition[18], while Brazil fosters the World Health Organization definition of *"those affecting less than 65 out of 100,000 individuals"*[19].

As RDs each individually affect a small number of people, they are "orphaned" by the pharmaceutical industry, which has promoted the use of the term "orphan disease" when referring to rare conditions[20]. To address the therapeutic gap in treatment options for orphan diseases, several countries have established mechanisms to encourage pharmaceutical companies to invest in, and manufacture, orphan drugs (ODs)[4 17 18 21].

Being termed an OD is a designation status issued by regulatory bodies to describe therapies that treat rare conditions[22 23]. Pharmaceutical companies that apply for such a designation are incentivised by these regulatory bodies if their therapy fulfils the designation criteria[22 23]. OD definitions vary beyond national boundaries. The UK[24], for instance, shares the same criterion as the European Medicines Agency, which is "*if the drug is intended for the diagnosis, prevention or treatment of a life-threatening or chronically and seriously debilitating condition affecting not more than 5 in 10,000 EU people or that it is unlikely that marketing the drug in the EU would generate sufficient benefit for the affected people and for the drug manufacturer to justify the investment"*[21]. Meanwhile, the FDA defines an OD as "*one intended for the treatment, prevention or diagnosis of a rare disease or condition, which is one that affects less than 200,000 persons in the US*" (which equates to approximately six cases per 10,000 population) *"or meets cost recovery provisions of the act*"[25 26]. Both definitions depend on the disease prevalence as a criterion for OD designation. In contrast, other countries may have more than one criterion,

For peer review only - http://bmjopen.bmj.com/site/about/guidelines.xhtml

including prevalence, for designating an OD. For instance, in Japan, to designate a drug as an orphan, the drug must meet these three criteria: the drug is used to treat an RD, there are no other treatments available in Japan, or the proposed drug is clinically superior to drugs already available on the Japanese market[21]. According to Section 16H of the Therapeutic Goods Regulations 1997 in Australia, ODs are defined as "*a medicine, vaccine, or in vivo diagnostic agent consider an orphan drug if it intended to treat, prevent or diagnose a rare disease; or must not be commercially viable to supply to treat, prevent, or diagnose another disease or condition*"[21 27].

As already mentioned, some countries foster an international RD definition by using other countries' criteria, which can be problematic due to differences in regional demographic, governmental, economic, and socio-cultural considerations[5]. For instance, consanguineous marriage (CM) is a major risk factor for congenital abnormalities and genetic diseases that are inherited in an autosomal recessive manner, resulting in some RDs[28]. In Saudi Arabia, there is no national definition for RD or OD, although CM represents 70% of the total marriages in the country[29 28 30 31]. Therefore, it could be considered improper for Saudi Arabia to espouse or adopt other countries' definitions.

This systematic review protocol describes an approach which will be utilised to review the published literature regarding the criteria used to define RDs and ODs from both qualitative and quantitative points of view, and to explore the rationale behind each criterion.

Review aims and objectives

Aim

This study aims to perform a systematic literature review to identify the criteria used to define RDs and ODs from both qualitative and quantitative perspectives.

Objectives

1. To identify the quantitative criteria used to define RDs and ODs
2. To identify the qualitative criteria used to define RDs and ODs
3. To explore the rationale behind the quantitative criteria used to define RDs and ODs
4. To explore the rationale behind the qualitative criteria used to define RDs and ODs

For peer review only - http://bmjopen.bmj.com/site/about/guidelines.xhtml

5. To explore the methodological characteristics that explain any heterogeneity in the results

Methods and analysis

This protocol is registered in PROSPERO (ID CRD42021252701) and will follow the preferred reporting items for systematic review and meta-analysis protocols (PRISMA-P, 2015)[32].

Inclusion criteria

The study will include any scientific publication of any design which discusses RDs and/or ODs and answer the research question: *What are the criteria to define Rare Diseases and Orphan Drugs globally?* Publications will be eligible for inclusion in the systematic review based on the framework known as the population, intervention, comparison, and outcome (PICO) framework[33]. There will be no restrictions on publication year or jurisdiction (North America, South America, Asia, Europe, Africa, Oceania, or the Middle East). We will include studies published in the English language reporting data for the general human population, with data extracted separately for children and adults if reported separately. The comparator element of the PICO framework will not be applicable for this study. Eligible articles will be analysed by searching for general definitions of RD and OD. These definitions will be categorised based on qualitative and quantitative criteria as an outcome of the review.

Exclusion criteria

RD due to infection will be excluded as some infections, such as tuberculosis, may be rare in wealthy countries, while being relatively common in poorer countries[34]. Exposure to toxic substances can cause RD, which will also be excluded from this review. For example, asbestos (fibrous silicate mineral) exposure can cause mesothelioma (a type of cancer that develops in the thin layer of tissue that covers the lungs and chest wall)[34]. Furthermore, rare forms of cancer will be excluded in our systematic review as there are discrepancies in rarity between countries that are typically captured by national cancer registries; for example, hepatocellular carcinoma is common in China (95.7/100,000 people) but is relatively uncommon in Canada (6.8/100,000 people)[35 36].

Search strategy

For peer review only - http://bmjopen.bmj.com/site/about/guidelines.xhtml

A systematic literature review will be conducted using Boolean Operator rules ('AND', 'OR', and 'NOT') with truncation to broaden the search terms to account for various word spellings and endings. The following search terms were discussed and validated by the research team along with a library information specialist, see Appendix (1): ultra-orphan disease; ultra-orphan drug; ultra-rare disease; orphan disease; orphan drug; orphan medicinal product; orphan product; orphan subset; orphan indication; orphan pharmaceutical product; orphan designation; neglected disease; rare and neglected disease; rare condition; rare disease; rare disorder; rare disability; rare medicinal technology; syndrome without a name; undiagnosed disease; life-threatening; debilitating; severe and intractable; highly specialized technology; very rare disease; low-frequency disease; pharmacological therapies of high complexity; priority review drugs; extremely rare disease; and orphan drug reimbursement system.

Publications will be retrieved from various databases, including PubMed, Medline, EMBASE, Scopus, Web of Science (science and social science citation index), and grey literature databases including GreyLit and OpenGrey, where additional legal terms (rare disease strategy, rare disease policy, rare disorder initiative, orphan disease declaration, rare disease national plan, orphan drug act, rare disease act, orphan drug regulation, orphan medical product decision, orphan drug directive, and orphan drug recommendation) will be used in the search strategy. To maximise literature saturation, this search will be complemented by reverse citation screening of relevant articles and forward citation searching. Databases will be searched from their commencement to 17th December 2021.

Study selection

Studies will be selected after publications are compiled, and duplicates will be removed. Two authors (GA and KK) will then independently conduct two rounds of screening of the titles and abstracts to assess initial eligibility based on the inclusion and exclusion criteria. The full text of both the publications that appear to meet the inclusion criteria, and those where there is insufficient information in the title and abstract to exclude the study, will be retrieved. Disagreements between the two individual reviewers will be resolved by a third reviewer (AJM) and all decisions will be documented in a Microsoft® Excel® spreadsheet. None of the reviewers will be blinded to the journal titles, the study authors, or the institutions.

For peer review only - http://bmjopen.bmj.com/site/about/guidelines.xhtml

236 **Data extraction**

237 The included articles will be summarised using a custom spreadsheet developed using the primary screening, quality assessment, and a data extraction web-based software platform tool, Covidence®[37]. The data will be extracted independently by two reviewers (GA and KK). Where there is missing or unreported data, or if additional details are required, the study's primary investigator (GA) will attempt to contact the study authors. The timeframe for a reply before the article is excluded based on the lack of requested information, will be three weeks.

243 The extracted data will include author(s) name(s), publication year, publication type, journal title, study design, organisation, geographical location, population, results of a study quality assessment, and the cited definitions of RD and OD. Moreover, the types of RD and OD criteria (qualitative or quantitative), their descriptor criteria, the rationale behind the definition, the status of the definition (developed or adopted), and whether RD and OD definitions have been considered (implicit or explicit) will also be recorded.

249 **Critical appraisal of studies**

250 Data extraction will be performed and each study's methodological rigour will be critically appraised by two authors (GA and KK) separately using customised forms based on the Joanna Briggs Institute (JBI) checklists[38]. The JBI's critical appraisal tools assist in assessing the trustworthiness, relevance, and results of published studies. Also, the grey literature will be evaluated and critically appraised using an AACODS checklist to assess its authority, accuracy, coverage, objectivity, date, and significance[39].

256 **Data synthesis**

257 Narrative synthesis will be undertaken using multiple studies to summarise and explain the current state of knowledge in relation to the systematic review's aim and objectives. An initial description of the results from each included article will be developed using a preliminary synthesis in the form of thematic and content analyses for qualitative descriptors, while descriptive analyses will be conducted in the form of frequencies and percentages for quantitative descriptors, and the results will be presented in a tabular format.

263 These data will be processed using various quantitative statistical and qualitative analysis approaches to investigate the similarities and differences between the studies, and to explore

For peer review only - http://bmjopen.bmj.com/site/about/guidelines.xhtml

265 the relationships within the data, with the aim of reporting key elements for defining RDs and ODs both qualitatively and quantitatively. The risk of bias will be assessed by considering the diversity of the study designs, populations, and outcomes.

Patient and Public Involvement

During the protocol development, there was no public or patient involvement. As the systematic review is focused on a secondary analysis of literature in the public domain, there will be no patient or public involvement in the systematic review.

Ethics and dissemination

Ethical committee approval is not required to conduct this systematic review, as no primary data will be collected; this systematic review is focused on a secondary analysis of the literature in the public domain. It is part of a PhD project at Queen's University Belfast, and the results will be presented at conferences and published as a thesis. We anticipate publication in a peer-reviewed journal. The results will guide stakeholders in defining RDs and ODs, and will inform policy decisions and future research.

Acknowledgement

The authors would like to thank Richard Fallis, a library information specialist, for his assistance in verifying the search terms and strategy to be used in this study.

Author contributions:

AJM and HA were responsible for research supervision. GA, AJM, and HA conceived, conceptualised, and designed this study. GA, KK, AJM, and HA will contribute to data acquisition and analysis. All authors contributed to the interpretation of results. GA drafted this manuscript. All the authors have reviewed the protocol for important intellectual content, approved the final version submitted for publication, and agreed to be accountable for all aspects of this research.

Funding

This systematic review is supported by the Medical Research Council Northern Ireland Executive in support of the Northern Ireland Genomic Medicine Centre through the Belfast Health and Social Care Trust award number (MC_PC_16018) and the Science Foundation Ireland and

For peer review only - http://bmjopen.bmj.com/site/about/guidelines.xhtml

Department for the Economy, Northern Ireland partnership award number (15/IA/3152). Princess Nourah University, Riyadh, Saudi Arabia, are supporting this study through a PhD scholarship with no grant number. These funders played no role in the development of this protocol.

Competing interests

The authors have no conflict of interests to declare.

Data sharing statement

GA, KK, and AJM confirm that relevant data are available within the article and its supplementary materials (study protocol and analysis plan).

For peer review only

For peer review only - http://bmjopen.bmj.com/site/about/guidelines.xhtml

References

1. Gorini F, Coi A, Mezzasalma L, et al. Survival of patients with rare diseases: a population-based study in Tuscany (Italy). *Orphanet J Rare Dis* 2021;16(1):275. doi: 10.1186/s13023-021-01907-0 [published Online First: 20210614]

2. Nguengang Wakap S, Lambert DM, Olry A, et al. Estimating cumulative point prevalence of rare diseases: analysis of the Orphanet database. *Eur J Hum Genet* 2020;28(2):165-73. doi: 10.1038/s41431-019-0508-0 [published Online First: 20190916]

3. Population matters. CURRENT WORLD POPULATION 2022 [Available from: https://populationmatters.org/population-numbers?gclid=EAIaIQobChMIjcjIlIO5-AIVA853Ch0PnwM_EAAYASAAEgIp7vD_BwE.

4. Chan AYL, Chan VKY, Olsson S, et al. Access and Unmet Needs of Orphan Drugs in 194 Countries and 6 Areas: A Global Policy Review With Content Analysis. *Value Health* 2020;23(12):1580-91. doi: 10.1016/j.jval.2020.06.020 [published Online First: 20201031]

5. Richter T, Nestler-Parr S, Babela R, et al. Rare Disease Terminology and Definitions-A Systematic Global Review: Report of the ISPOR Rare Disease Special Interest Group. *Value Health* 2015;18(6):906-14. doi: 10.1016/j.jval.2015.05.008 [published Online First: 20150818]

6. Steven Simoens DC, Marc Dooms, Eline Picavet. Orphan Drugs for Rare Diseases. *Drugs* 2012(72):1437–43 doi: https://doi.org/10.2165/11635320-000000000-00000

7. Rode J. Rare Diseases: understanding this Public Health Priority: Eurordis; 2005 [Available from: https://www.eurordis.org/IMG/pdf/princeps_document-EN.pdf2021.

8. Melissa Conrad Stöppler M. Medical Definition of Prevalence 2021 [Available from: https://www.medicinenet.com/prevalence/definition.htm.

9. Leadley RM, Lang S, Misso K, et al. A systematic review of the prevalence of Morquio A syndrome: challenges for study reporting in rare diseases. *Orphanet J Rare Dis* 2014;9:173. doi: 10.1186/s13023-014-0173-x [published Online First: 20141118]

10. Alpsoy E, Akman-Karakas A, Uzun S. Geographic variations in epidemiology of two autoimmune bullous diseases: pemphigus and bullous pemphigoid. *Arch Dermatol Res* 2015;307(4):291-8. doi: 10.1007/s00403-014-1531-1 [published Online First: 20150115]

For peer review only - http://bmjopen.bmj.com/site/about/guidelines.xhtml

11. Lord B. RS, Vaughan G., Mairi G. The UK Rare Diseases Framework 2021 [Available from: https://www.gov.uk/government/publications/uk-rare-diseases-framework/the-uk-rare-diseases-framework.

12. Regulation (EC) No 141/2000 of the European Parliament and of the Council of 16 December 1999 on orphan medicinal products. 1999 [Available from: http://eur-lex.europa.eu/smartapi/cgi/sga_doc?smartapi!celexapi!prod!CELEXnumdoc&lg=en&numdoc=32000R0141&model=guichett).

13. Health NIo. PUBLIC LAW 107–280—NOV. 6, 2002. In: 2002 RDA, ed., 2002.

14. Health. NIo. Public Law 97–414 97th Congress. In: Health NIo, ed., 1983.

15. US Code. 21 USC 360ee: Grants and contracts for development of drugs for rare diseases and conditions 2022, July 1 [Available from: https://uscode.house.gov/view.xhtml?req=granuleid:USC-prelim-title21-section360ee&num=0&edition=prelim.

16. U.S. Food & Drug Adminstration. DESIGNATION OF DRUGS FOR RARE DISEASES OR CONDITIONS SEC. 526 OF THE FEDERAL FOOD, DRUG, AND COSMETIC ACT [21 USC 360bb]. 2018 [Available from: https://www.fda.gov/industry/designating-orphan-product-drugs-and-biological-products/orphan-drug-act-relevant-excerpts.

17. Stawowczyk E, Malinowski KP, Kawalec P, et al. Reimbursement Status and Recommendations Related to Orphan Drugs in European Countries. *Front Pharmacol* 2019;10:1279. doi: 10.3389/fphar.2019.01279 [published Online First: 20191127]

18. Arnold RJ, Bighash L, Bryon Nieto A, et al. The role of globalization in drug development and access to orphan drugs: orphan drug legislation in the US/EU and in Latin America. *F1000Res* 2015;4:57. doi: 10.12688/f1000research.4268.1 [published Online First: 20150227]

19. Passos-Bueno MR, Bertola D, Horovitz DD, et al. Genetics and genomics in Brazil: a promising future. *Mol Genet Genomic Med* 2014;2(4):280-91. doi: 10.1002/mgg3.95 [published Online First: 2014/08/01]

20. Rare and Orphan Lung Disease European Lung Foundation2021 [Available from: https://europeanlung.org/en/information-hub/lung-conditions/rare-and-orphan-lung-disease/.

For peer review only - http://bmjopen.bmj.com/site/about/guidelines.xhtml

21. Sharma A, Jacob A, Tandon M, et al. Orphan drug: Development trends and strategies. *J Pharm Bioallied Sci* 2010;2(4):290-9. doi: 10.4103/0975-7406.72128 [published Online First: 2010/12/25]

22. European Medicines Agency. Orphan designation 2022 [Available from: https://www.ema.europa.eu/en/glossary/orphan-designation accessed 06/01/2022.

23. U.S. Food and Drugs Administration. Drug Designation 12/17/2021 [Available from: https://www.fda.gov/animal-veterinary/minor-useminor-species/drug-designation accessed 06/01/2022.

24. Agency MaHpR. Guidance Orphan medicinal products 31 December 2020 [Available from: https://www.gov.uk/guidance/orphan-medicinal-products-in-great-britain.

25. Developing Products for Rare Diseases & Conditions US Food and Drug Adminstration20 December 2018. Retrieved 28 December 2019 [Available from: https://www.fda.gov/industry/developing-products-rare-diseases-conditions.

26. Hall AK, Carlson MR. The current status of orphan drug development in Europe and the US. *Intractable Rare Dis Res* 2014;3(1):1-7. doi: 10.5582/irdr.3.1 [published Online First: 2014/10/25]

27. Herkes GK. Orphan drugs in Australia. *Expert Opinion on Orphan Drugs* 2016;4(12):1195-97. doi: 10.1080/21678707.2016.1257383

28. Alahdal H, Alshanbari H, Almazroa H, et al. Consanguinity, awareness, and genetic disorders among female university students in Riyadh, Saudi Arabia. *Journal of Biochemical and Clinical Genetics* 2021;4(1):27-34. doi: 10.24911/JBCGenetics/183-1601264923

29. Al-Gazali L, Hamamy H, Al-Arrayad S. Genetic disorders in the Arab world. *BMJ* 2006;333(7573):831-4. doi: 10.1136/bmj.38982.704931.AE

30. AbdulAzeez S, Al Qahtani NH, Almandil NB, et al. Genetic disorder prenatal diagnosis and pregnancy termination practices among high consanguinity population, Saudi Arabia. *Sci Rep* 2019;9(1):17248. doi: 10.1038/s41598-019-53655-8 [published Online First: 20191121]

31. Mahboub SM, Alsaqabi AA, Allwimi NA, et al. Prevalence and pattern of consanguineous marriage among educated married individuals in Riyadh. *J Biosoc Sci* 2020;52(5):768-75. doi: 10.1017/S0021932019000786 [published Online First: 20191129]

For peer review only

For peer review only - http://bmjopen.bmj.com/site/about/guidelines.xhtml

32. Moher D, Shamseer L, Clarke M, et al. Preferred reporting items for systematic review and meta-analysis protocols (PRISMA-P) 2015 statement. *Syst Rev* 2015;4:1. doi: 10.1186/2046-4053-4-1 [published Online First: 20150101]

33. Tove FaberFrandsen MFN, Christina LouiseLindhardt, Mette BrandtEriksen. Using the full PICO model as a search tool for systematic reviews resulted in lower recall for some PICO elements. *Journal of Clinical Epidemiology* 2020

34. Development IoMUCoARDRaOP. Rare Diseases and Orphan Products: Accelerating Research and Development. Washington (DC): National Academies Press (US), 2010.

35. McGlynn KA, London WT. Epidemiology and natural history of hepatocellular carcinoma. *Best Pract Res Clin Gastroenterol* 2005;19(1):3-23. doi: 10.1016/j.bpg.2004.10.004 [published Online First: 2005/03/11]

36. De P, Dryer D, Otterstatter MC, et al. Canadian trends in liver cancer: a brief clinical and epidemiologic overview. *Curr Oncol* 2013;20(1):e40-3. doi: 10.3747/co.20.1190 [published Online First: 2013/02/28]

37. Innovation VH. Covidence systematic review software Melbourne, AustraliaFebruary 4, 2019 [Available from: https://www.covidence.org/2021.

38. Institute JB. Critical appraisal tools 2020 [Available from: https://jbi.global/critical-appraisal-tools.

39. Tyndall J. AACODS Checklist. 2010 [Available from: https://dspace.flinders.edu.au/xmlui/bitstream/handle/2328/3326/AACODS_Checklist.pdf?sequence=4&isAllowed=y.

For peer review only - http://bmjopen.bmj.com/site/about/guidelines.xhtml

Appendix 1: Search strategy

Research question: What are the criteria to define Rare Diseases and Orphan Drugs globally?

	PubMed	Medline	Embase	Scopus	WOS
Concept 1 (Criteria)	Criteria [All Fields] OR Standard*[All Fields] OR classification [All Fields] OR Measure*[All Fields] OR Condition*[All Fields] OR Principle*[All Fields] OR Requirement*[All Fields] OR Scale*[All Fields] OR Parameter*[All Fields] OR Indicator*[All Fields] OR Norm*[All Fields]	(Criteria or Standard* or classification or Measure* or Condition* or Principle* or Requirement* or Scale* or Parameter* or Indicator* or Norm*).mp. [mp=title, abstract, original title, name of substance word, subject heading word, floating sub-heading word, keyword heading word, organism supplementary concept word, protocol supplementary concept word, rare disease supplementary concept word, unique identifier, synonyms]	(Criteria or Standard* or classification or Measure* or Condition* or Principle* or Requirement* or Scale* or Parameter* or Indicator* or Norm*).mp. [mp=title, abstract, heading word, drug trade name, original title, device manufacturer, drug manufacturer, device trade name, keyword, floating subheading word, candidate term word]	TITLE-ABS-KEY (criteria OR standard* OR classification OR measure* OR condition* OR principle* OR requirement* OR scale* OR parameter* OR indicator* OR norm*)	ALL FIELDS: (criteria OR standard* OR classification OR measure* OR condition* OR principle* OR requirement* OR scale* OR parameter* OR indicator* OR norm*) Timespan: All years. Indexes: SCI-EXPANDED, SSCI, A&HCI, CPCI-S, CPCI-SSH, ESCI.
Concept 2 (Define)	Defin*[All Fields] OR Mean*[All Fields] OR Description [All Fields] OR Character*[All Fields] OR Explan*[All Fields] OR delineate [All Fields] OR detail [All Fields] OR interpret[All Fields] OR determine[All Fields] OR elucidate[All Fields] OR illustrate[All Fields] OR exemplify[All Fields]	(Defin* or Mean* or Description or Character* or Explan* or delineate or detail or interpret or determine or elucidate or illustrate or exemplify).mp. [mp=title, abstract, original title, name of substance word, subject heading word, floating sub-heading word, keyword heading word, organism supplementary concept word, protocol supplementary concept word, rare disease supplementary concept word, unique identifier, synonyms]	(Defin* or Mean* or Description or Character* or Explan* or delineate or detail or interpret or determine or elucidate or illustrate or exemplify).mp. [mp=title, abstract, heading word, drug trade name, original title, device manufacturer, drug manufacturer, device trade name, keyword, floating subheading word, candidate term word]	TITLE-ABS-KEY (defin* OR mean* OR description OR character* OR explan* OR delineate OR detail OR interpret OR determine OR elucidate OR illustrate OR exemplify)	ALL FIELDS: (defin* OR mean* OR description OR character* OR explan* OR delineate OR detail OR interpret OR determine OR elucidate OR illustrate OR exemplify) Timespan: All years. Indexes: SCI-EXPANDED, SSCI, A&HCI, CPCI-S, CPCI-SSH, ESCI.
Concept 3 (Rare Disease(s)	"Rare Diseases"[Mesh] OR “Orphan disease*”[All Fields] OR “Rare condition*”[All Fields] OR “Rare disorder*”[All Fields] OR “Rare disability*”[All Fields] OR “Neglected disease*”[All Fields] OR “Undiagnosed disease*”[All Fields] OR “Low-frequency disease*”[All Fields] OR “life-threatening disease*”[All Fields] OR “debilitating disease*”[All Fields] OR “severe disease*”[All Fields] OR “intractable disease*”[All Fields]	(Orphan disease* or Rare condition* or Rare disorder* or Rare disability* or Neglected disease* or Undiagnosed disease* or Low-frequency disease* or life-threatening disease* or debilitating disease* or severe disease* or intractable disease* or Rare Disease*).mp. [mp=title, abstract, original title, name of substance word, subject heading word, floating sub-heading word, keyword heading word, organism supplementary concept word, protocol supplementary concept word, rare disease supplementary concept word, unique identifier, synonyms]	(Orphan disease* or Rare condition* or Rare disorder* or Rare disability* or Neglected disease* or Undiagnosed disease* or Low-frequency disease* or life-threatening disease* or debilitating disease* or severe disease* or intractable disease* or Rare Disease*).mp. [mp=title, abstract, heading word, drug trade name, original title, device manufacturer, drug manufacturer, device trade name, keyword, floating subheading word, candidate term word]	TITLE-ABS-KEY ("Orphan disease*" OR "Rare condition*" OR "Rare disorder*" OR "Rare disability*" OR "Neglected disease*" OR "Undiagnosed disease*" OR "Low-frequency disease*" OR "life-threatening disease*" OR "debilitating disease*" OR "severe disease*" OR "intractable disease*" OR "Rare Disease*")	ALL FIELDS: ("Orphan disease*" OR “Rare condition*” OR “Rare disorder*” OR “Rare disability*” OR “Neglected disease*” OR “Undiagnosed disease*” OR “Low-frequency disease*” OR “life-threatening disease*” OR “debilitating disease*” OR “severe disease*” OR “intractable disease*” OR “Rare Disease*”) Timespan: All years. Indexes: SCI-EXPANDED, SSCI, A&HCI, CPCI-S, CPCI-SSH, ESCI.

For peer review only

For peer review only - http://bmjopen.bmj.com/site/about/guidelines.xhtml

Concept 4 (Orphan Drug(s))	"Orphan Drug Production"[Mesh] OR “Orphan medicinal product*” [All Fields] OR “Orphan product*”[All Fields] OR “Orphan subset*”[All Fields] OR “Orphan indication*”[All Fields] OR “Highly specialized technolog*”[All Fields] OR “Priority review drug*”[All Fields] OR "Orphan Drug*"[All Fields]	(Orphan medicinal product* or Orphan product* or Orphan subset* or Orphan indication* or Highly specialized technolog* or Priority review drug* or Orphan Drug* or Orphan Drug Production*).mp. [mp=title, abstract, original title, name of substance word, subject heading word, floating sub-heading word, keyword heading word, organism supplementary concept word, protocol supplementary concept word, rare disease supplementary concept word, unique identifier, synonyms]	(Orphan medicinal product* or Orphan product* or Orphan subset* or Orphan indication* or Highly specialized technolog* or Priority review drug* or Orphan Drug* or Orphan Drug Production*).mp. [mp=title, abstract, heading word, drug trade name, original title, device manufacturer, drug manufacturer, device trade name, keyword, floating subheading word, candidate term word]	TITLE-ABS-KEY ("Orphan medicinal product*" OR "Orphan product*" OR "Orphan subset*" OR "Orphan indication*" OR "Highly specialized technolog*" OR "Priority review drug*" OR "Orphan Drug Production*" OR "Orphan Drug*")	ALL FIELDS: ("Orphan medicinal product*" OR "Orphan product*" OR "Orphan subset*" OR "Orphan indication*" OR "Highly specialized technolog*" OR "Priority review drug*" OR "Orphan Drug Production*" OR "Orphan Drug*") Timespan: All years. Indexes: SCI-EXPANDED, SSCI, A&HCI, CPCI-S, CPCI-SSH, ESCI.
Total	(((Criteria [All Fields] OR Standard*[All Fields] OR classification [All Fields] OR Measure*[All Fields] OR Condition*[All Fields] OR Principle*[All Fields] OR Requirement*[All Fields] OR Scale*[All Fields] OR Parameter*[All Fields] OR Indicator*[All Fields] OR Norm*[All Fields]) OR (Defin*[All Fields] OR Mean*[All Fields] OR Description [All Fields] OR Character*[All Fields] OR Explan*[All Fields] OR delineate [All Fields] OR detail [All Fields] OR interpret[All Fields] OR determine[All Fields] OR elucidate[All Fields] OR illustrate[All Fields] OR exemplify[All Fields])) AND ("Rare Diseases"[Mesh] OR "Orphan disease*"[All Fields] OR "Rare condition*"[All Fields] OR "Rare disorder*"[All Fields] OR "Rare disability*"[All Fields] OR "Neglected disease*"[All Fields] OR "Undiagnosed disease*"[All Fields] OR "Low-frequency disease*"[All Fields] OR "life-threatening disease*"[All Fields] OR "debilitating disease*"[All Fields] OR "severe disease*"[All Fields] OR "intractable disease*"[All Fields])) AND ("Orphan Drug Production"[Mesh] OR "Orphan medicinal product*" [All Fields] OR "Orphan product*"[All Fields] OR "Orphan subset*"[All Fields] OR "Orphan indication*"[All Fields] OR "Highly specialized technolog*"[All Fields] OR "Priority review drug*"[All Fields] OR "Orphan Drug*"[All Fields])	1 OR 2 And 3 and 4	1 OR 2 And 3 and 4	(TITLE-ABS-KEY (criteria OR standard* OR classification OR measure* OR condition* OR principle* OR requirement* OR scale* OR parameter* OR indicator* OR norm*)) OR (TITLE-ABS-KEY (defin* OR mean* OR description OR character* OR explan* OR delineate OR detail OR interpret OR determine OR elucidate OR illustrate OR exemplify)) AND (TITLE-ABS-KEY ("Orphan disease*" OR "Rare condition*" OR "Rare disorder*" OR "Rare disability*" OR "Neglected disease*" OR "Undiagnosed disease*" OR "Low-frequency disease*" OR "life-threatening disease*" OR "debilitating disease*" OR "severe disease*" OR "intractable disease*" OR "Rare Disease*")) AND (TITLE-ABS-KEY ("Orphan medicinal product*" OR "Orphan product*" OR "Orphan subset*" OR "Orphan indication*" OR "Highly specialized technolog*" OR "Priority review drug*" OR "Orphan Drug Production*"))	#7 AND #6 AND #5 Timespan: All years. Indexes: SCI-EXPANDED, SSCI, A&HCI, CPCI-S, CPCI-SSH, ESCI. ...Less

For peer review only

For peer review only - http://bmjopen.bmj.com/site/about/guidelines.xhtml

1

PRISMA-P 2015 Checklist

This checklist has been adapted for use with protocol submissions to *Systematic Reviews* from Table 3 in Moher D et al: Preferred reporting items for systematic review and meta-analysis protocols (PRISMA-P) 2015 statement. *Systematic Reviews* 2015 **4**:1

Section/topic	#	Checklist item	Information reported		Line number(s)
			Yes	No	
**ADMINISTRATIVE INFORMATION**					
**Title**					
Identification	1a	Identify the report as a protocol of a systematic review	☒	☐	3
Update	1b	If the protocol is for an update of a previous systematic review, identify as such	☐	☒	n/a
**Registration**	2	If registered, provide the name of the registry (e.g., PROSPERO) and registration number in the Abstract	☒	☐	61, 174
**Authors**					
Contact	3a	Provide name, institutional affiliation, and e-mail address of all protocol authors; provide physical mailing address of corresponding author	☒	☐	4-22
Contributions	3b	Describe contributions of protocol authors and identify the guarantor of the review	☒	☐	338-344
**Amendments**	4	If the protocol represents an amendment of a previously completed or published protocol, identify as such and list changes; otherwise, state plan for documenting important protocol amendments	☐	☒	n/a
**Support**					
Sources	5a	Indicate sources of financial or other support for the review	☒	☐	346-350
Sponsor	5b	Provide name for the review funder and/or sponsor	☒	☐	346-350
Role of sponsor/funder	5c	Describe roles of funder(s), sponsor(s), and/or institution(s), if any, in developing the protocol	☒	☐	350
**INTRODUCTION**					
**Rationale**	6	Describe the rationale for the review in the context of what is already known	☒	☐	151-161
**Objectives**	7	Provide an explicit statement of the question(s) the review will address with reference to participants, interventions, comparators, and outcomes (PICO)	☒	☐	167-172 , 178 - 180
**METHODS**					

BioMed Central The Open Access Publisher

Section/topic	#	Checklist item	Information reported		Line number(s)
			Yes	No	
**Eligibility criteria**	8	Specify the study characteristics (e.g., PICO, study design, setting, time frame) and report characteristics (e.g., years considered, language, publication status) to be used as criteria for eligibility for the review	☒	☐	176-187
**Information sources**	9	Describe all intended information sources (e.g., electronic databases, contact with study authors, trial registers, or other grey literature sources) with planned dates of coverage	☒	☐	199- 214
**Search strategy**	10	Present draft of search strategy to be used for at least one electronic database, including planned limits, such that it could be repeated	☒	☐	361
STUDY RECORDS					
Data management	11a	Describe the mechanism(s) that will be used to manage records and data throughout the review	☒	☐	50, 222, 225
Selection process	11b	State the process that will be used for selecting studies (e.g., two independent reviewers) through each phase of the review (i.e., screening, eligibility, and inclusion in meta-analysis)	☒	☐	217, 218
Data collection process	11c	Describe planned method of extracting data from reports (e.g., piloting forms, done independently, in duplicate), any processes for obtaining and confirming data from investigators	☒	☐	216,227-229
**Data items**	12	List and define all variables for which data will be sought (e.g., PICO items, funding sources), any pre-planned data assumptions and simplifications	☒	☐	230-235
**Outcomes and prioritization**	13	List and define all outcomes for which data will be sought, including prioritization of main and additional outcomes, with rationale	☒	☐	230-235
**Risk of bias in individual studies**	14	Describe anticipated methods for assessing risk of bias of individual studies, including whether this will be done at the outcome or study level, or both; state how this information will be used in data synthesis	☒	☐	253
DATA					
**Synthesis**	15a	Describe criteria under which study data will be quantitatively synthesized	☒	☐	92, 98, 186,187
	15b	If data are appropriate for quantitative synthesis, describe planned summary measures, methods of handling data, and methods of combining data from studies, including any planned exploration of consistency (e.g., I^2, Kendall's tau)	☒	☐	52, 53, 250-253
	15c	Describe any proposed additional analyses (e.g., sensitivity or subgroup analyses, meta-regression)	☐	☒	n/a
	15d	If quantitative synthesis is not appropriate, describe the type of summary planned	☒	☐	186, 247, 250 - 253
**Meta-bias(es)**	16	Specify any planned assessment of meta-bias(es) (e.g., publication bias across studies, selective reporting within studies)	☐	☒	n/a

For peer review only

BioMed Central
The Open Access Publisher

3

Section/topic	#	Checklist item	Information reported		Line number(s)
			Yes	No	
**Confidence in cumulative evidence**	17	Describe how the strength of the body of evidence will be assessed (e.g., GRADE)	☒	☐	237-242

For peer review only

BioMed Central
The Open Access Publisher
